# The Economic Context of Higher Education Expansion: Race, Gender, and Household Finances Across Cohorts and Generations

**DOI:** 10.1007/s10834-023-09918-8

**Published:** 2023-07-01

**Authors:** Natasha Quadlin, Jordan A. Conwell, Shiva Rouhani

**Affiliations:** 1 Department of Sociology, University of California, Los Angeles, 264 Haines Hall, 375 Portola Plaza, Los Angeles, CA 90095, USA; 2 University of Texas at Austin, Austin, USA

**Keywords:** Race, Gender, Higher education, Income and wealth inequality, Student loan debt

## Abstract

This article assesses how the economic context of higher education expansion since the mid-20th century has shaped families’ financial lives—in terms of income and wealth/debt—as well as how these trends have differed for Black and White women and men. We use data from the NLSY-79 (comprising trailing-edge Baby Boomers) and NLSY-97 (comprising early Millennials) to show how academically similar students in these two cohorts fared in terms of educational attainment, household income, household wealth, and total student debt accrued by age 35. While we discuss findings across race-gender groups, our results call attention to the education-related economic disadvantages faced by Black women that have accelerated across cohorts. Over time, Black women’s educational attainment has increased substantially, and high-achieving Black women, in particular, have become uniquely likely to progress beyond the BA. But while high-achieving Black women have made many advances in higher education, they also have become more likely than similarly high-achieving White men, White women, and Black men to have zero or negative wealth at the household level, and to accrue student debt for themselves and for their children. Our findings demonstrate that the costs of expanded access to credit for higher education have not been borne equally across race, gender, and achievement, and that these patterns have multigenerational financial consequences for college attendees and their families.

## Introduction

Rates of college attendance and completion have increased markedly over the past 50 years (Bowen et al., 2009; DiPrete & Buchmann, 2013; Quadlin & Powell, 2022). Because higher education is an important predictor of employment status, occupational prestige, wages, and other economic outcomes (Carnevale et al., 2013; Hout, 2011; Lemieux, 2006), college attendance theoretically should be a boon to households’ financial standing in one generation as well as families’ economic mobility between generations. But at the same time that college attendance has become more common, the cost of attending college has increased dramatically, alongside the expansion of credit and student debt (Addo et al., 2016; Dwyer, 2018; Dwyer et al., 2012; Houle, 2014a; Houle & Addo, 2022; Zaloom, 2019). This ultimately creates liabilities for individuals hoping to enhance their human capital with a college credential—especially those who aspire to graduate and professional degrees, as levels of student debt often are highest among these individuals (Pyne & Grodsky, 2020).

These trends are consequential in the context of racial and intersectional (here, race-gender) inequality. Trends in higher education expansion are nested within historical and continued structural racism that advantages Whites and disadvantages Blacks and other populations of color (Bonilla-Silva, 1997). Black students are more likely to complete college than they once were, and this is especially the case for Black women, whose rates of college completion have exceeded Black men’s since 1920s birth cohorts (McDaniel et al., 2011). However, Black college graduates face multipronged disadvantages that threaten their ability to attain high income and wealth. Black college graduates face racial discrimination in the labor market, which suppresses their wages and occupational standing (Gaddis, 2015; Nunley .:(et al., 2015); they accrue disproportionately high amounts of student debt with less favorable repayment terms than that of their White peers (Houle & Addo, 2019, 2022; Quadlin & Conwell, 2021); and they have much less family wealth than comparable White students, giving them a weaker safety net in young adulthood (Massey et al., 2003). Because Black women’s participation in higher education is systematically higher than Black men’s (especially among Black women with relatively strong academic preparation, as we will discuss throughout this article), these are not simply racialized patterns of disadvantage, but instead they are deeply intersectional (Crenshaw, 1989), with Black women being more likely than Black men to be exposed.

Although these are not new observations, little research explicitly brings these trends in conversation with each other to understand the broader economic context of educational expansion for Black and White women and men and their families. In this paper, we examine how students in two distinct cohorts with similar academic credentials fared in terms of their educational attainment, household income, household wealth, and student debt. This approach is informed by prior research on the unequal economic origins of high-achieving students across race-gender groups (Quadlin & Conwell, 2021). As college has become a normative stage in the life course, we might expect that students’ chances of attending college have been lifted, especially among those with strong academic credentials. Yet, the economic consequences of such exposure have been uneven. Specifically, we make comparisons across race-gender groups among students with similar standardized test scores, as we will discuss throughout. Such test scores are commonly used as shorthand for student “ability”; and while we and many others object to this characterization because it overlooks the strong influence of socioeconomic status and other aspects of social (dis)advantage on such measures (Riegle-Crumb et al., 2019), our analyses also underscore that the life course economic payoffs to high test scores (and, by extension, their relatively high probability of college attendance) are unequal across race-gender groups.

The data come from two nationally representative datasets representing cohorts with distinct experiences in higher education and the labor market. Respondents in the National Longitudinal Survey of Youth, 1979 Cohort (NLSY-79) are trailing-edge Baby Boomers, born between 1957 and 1964. Respondents in the National Longitudinal Survey of Youth, 1997 Cohort (NLSY-97) are early Millennials, born between 1980 and 1984. We use these datasets to show how Black and White women and men with equivalent test scores fared in these two cohorts in terms of their educational and household economic attainment, thus broadly showing how changes in historical context correspond with shifts in intersectional economic inequality among those with comparable measured college readiness.

### Higher Education Expansion and Implications for Race-Gender Inequality

As much research has shown, college enrollment and completion in the U.S. have increased dramatically over the past several decades. In 1970, when the NLSY-79 respondents were in elementary and middle school, 11 percent of adults ages 25 and older had attained a bachelor’s degree. By 2017, that figure had increased to 34 percent (United States Census Bureau, 2017)—not to mention the sizeable number of Americans who start but do not complete a bachelor’s degree, estimated at about 40 percent of those who ever enroll in bachelor’s degree-granting institutions (Snyder et al., 2019).

At the same time that enrollment in higher education has increased, so too has the cost of attending college. Students in 1987 could attend a public 4-year university, including room and board, for about $9000/year in 2017 dollars. Today, that figure has increased to about $21,000/year, although the sticker prices at some elite private institutions approach $80,000/year (College Board, 2019). The reasons for these price increases are complex and ultimately outside the scope of this article, but social scientists have pointed to state disinvestment, coupled with a reliance on individual financing and debt, as some of the major social forces contributing to these trends (Houle & Addo, 2022; Quadlin & Powell, 2022; Zaloom, 2019).

Prior research on how college attendance fits into the economic life course has unevenly attended to consequential interrelationships between income, wealth, and debt. A prominent literature in the social sciences, for example, focuses on identifying the causal “value-added” of college attendance, especially colleges of a given level of prestige or selectivity (e.g., Dale & Krueger, 2002). These studies tend to focus on individual income as the primary outcome variable, consistent with the perspective that college is an investment in one’s own human capital (Becker, 1964). By comparison, the literature on student debt understandably has been more even-handed about both the promise and perils of investing in one’s education through access to credit. Dwyer and colleagues (2012), for example, describe student debt as a “double-edged sword,” or an important facilitator of opportunity as well as a great liability. Given that these liabilities are unevenly borne across social groups, as we discuss further below, our main analyses consider the patterning of educational attainment, income, and wealth alongside the patterning of student debt.

In the case of student debt, for the older cohort in our study (i.e., the NLSY-79), we can incorporate intergenerational measures that capture student debt for oneself as well as one’s children. Parent loans, including but not limited to PLUS loans, are often overlooked in research, but are a potentially meaningful source of intergenerational burden (Cepa, 2021). Such loans require parents to take on economic liabilities at a potentially precarious time; while many of these parents are prime-aged workers, and thus are in a relatively good position to repay debt, they also may be near retirement and thus not ideally equipped to take on a large and/or long-term economic investment (Fletcher et al., 2020). To the extent that parent loans are being disbursed unequally across race-gender groups, this would indicate inequality in terms of who is being further stretched at a potentially vulnerable point in the life course.

We focus on how these measures have unfolded for Black and White women and men. As background, much recent research has discussed two major trends with respect to race and gender in higher education. The first is racial disparities in student debt (see Houle & Addo, 2022). Although Black students are more likely to enroll in and complete college than they once were, the average Black college student hails from a far less economically advantaged family than that of their White peers. Black students are more likely to accrue debt than White students (Houle, 2014b); among those with debt, Black students owe $5000–10,000 more than White students on average (Jackson & Reynolds, 2013). Black students also are more likely than White students to default on their loans (Gross et al., 2009; Scott-Clayton & Li, 2016) and experience financial distress because of their loans (Martin & Dwyer, 2021). In fact, scholars recently have argued that Black students have been incorporated in higher education under terms of “predatory inclusion,” such that they have access to postsecondary institutions, but in a way that leaves them financially vulnerable (Seamster & Charron-Chénier, 2017).

The second trend of interest is the “rise of women,” that is, women’s steady gains in higher education that led to a reversal of the gender gap in college enrollment and completion in the mid-1980s (Buchmann & DiPrete, 2006; DiPrete & Buchmann, 2013; Goldin et al., 2006). While much research discusses this trend without regard to race, others have noted that the reversal of the gender gap that occurred in the 1980s was mostly driven by White men and women. Among Black men and women, women’s advantage emerged much earlier than this; Black women’s rates of college enrollment and completion exceeded Black men’s starting with 1920s birth cohorts, and their advantage continued to grow through the modern era (DiPrete & Buchmann, 2013; McDaniel et al., 2011). Previous research focused on the set of structurally based disadvantages facing Black boys and men points to the group’s higher likelihood of being exposed to factors such as exclusionary school discipline, policing, and carceral facilities as factors limiting the group’s average educational trajectories. In the education space, for example, recent experimental research demonstrates that teachers are more likely to punish Black boys than White boys for identical misbehavior (Owens, 2022).

Taking these two trends together, we highlight Black women as a demographic group with structural overexposure to educational debt—what we might characterize as a perverse consequence of their educational persistence. Prior research has especially highlighted economically disadvantaged Black women’s predatory inclusion in higher education via for-profit institutions. Cottom (2017) shows how these institutions target women’s desire for financial independence and encourage them to take on much more debt than they can reasonably handle, only to offer them weak training and poor economic returns. Others have considered how the financial burdens of higher education tend to fall disproportionately on Black women who are relatively advantaged. Research shows that Black girls with the strongest academic credentials in high school—i.e., those who are most likely to enter 4-year institutions and potentially continue on to graduate school—have far fewer economic resources than their similarly high-achieving peers in other race-gender groups, including White boys, White girls, *and* Black boys (Quadlin & Conwell, 2021). Thus, as a result of Black women’s and girls’ tendency to academically out-perform their economic circumstances, they may be disproportionately likely to enter costly institutions with few financial resources.

We systematically consider how these generational and race-gender patterns differ by students’ measured academic test scores in adolescence. From a theoretical standpoint, these test scores should be unambiguously positively related to a range of attainment outcomes. The status attainment model, for example, included achievement as a key predictor of educational and occupational attainment (Sewell et al., 1969, 1970; also see Blau & Duncan, 1967). Although the relationship between youth test scores and adult economic success may be straightforward for many individuals, such models do not account for the high cost of higher education, nor do they consider potential disparate returns to education across race-gender groups. We thus consider our outcomes across the range of test scores, guided by empirical findings on Black women’s selection into higher education, as well as their economic origins and outcomes. As many scholars have demonstrated, standardized test scores measure both ability and opportunity (Conwell, 2021; Neal & Johnson, 1996; Riegle-Crumb et al., 2019), with the latter in our minds including how a child’s family, school, neighborhood, healthcare, and other contexts facilitate or inhibit the expression of their innate capabilities via academic tests. Despite these caveats, we follow other researchers in noting these measures are also analytically useful because they are measured comparably across cohorts and are strongly correlated with college attendance and completion and later life course financial outcomes. As we demonstrate, the educational and economic payoffs to high test scores vary substantially across cohorts and race-gender groups.

### Data, Measures, and Methods Using Two Strategically Timed Cohorts

We use data from two nationally representative datasets representing two U.S. cohorts that have had very different experiences in higher education and in their access to and use of credit. The NLSY-79 is a survey of 12,686 respondents born between 1957 and 1965 (we refer to them as “Baby Boomers”). When they were first interviewed in the base year of 1979, they were 14–22 years old. Respondents were interviewed annually through 1994 and biennially since then. The NLSY-97 is a survey of 8984 respondents born between 1980 and 1984 (we refer to them as “Millennials”). When they were first interviewed in the base year of 1997, they were 12–16 years old. Respondents were interviewed annually through 2011 and biennially since then.

Although these surveys were fielded during different time periods, they share a focus on youth’s experiences in education and the labor market, and they boast high retention rates well into respondents’ adult years. The two surveys also contain many of the same or comparable measures, making them well-suited for our cross-cohort comparison of educational attainment and economic outcomes. We restrict both samples to four race-gender groups that also constitute our main predictor variables: White men, White women, Black men, and Black women. The main analyses also stratify respondents according to standardized tests that NLSY administered in both cohorts’ base year. In the NLSY-79, this is the Armed Forces Qualifying Test (AFQT), and in the NLSY-97, this is the Armed Services Vocational Aptitude Battery (ASVAB), although we use only the academic subtests of the ASVAB in order to make this measure functionally equivalent to the AFQT. Descriptive statistics are shown in Table 1. The analytic sample includes all White and Black respondents with complete data on the AFQT/ASVAB and our economic outcomes of interest (discussed below), whether they attended college or not.^1^

We use four sets of outcome variables that gauge respondents’ educational attainment and economic standing, which we generally measure when respondents are age 35, as discussed further below. *Educational attainment* is measured as respondents’ highest level of education completed. *Household income* is measured as total net family income from the previous calendar year. *Household wealth* is a composite measure of all assets minus all debts. We assess wealth in dollar amounts as well as, importantly, those who have no/negative wealth versus positive wealth (0/1). Then, given our focus on the economic consequences of higher education expansion as well as inequality across the life course, we constructed measures of *cumulative student loans accrued*. These include student debt accrued for the respondent’s own education (available for both cohorts) and student loans accrued for the respondent’s children’s education (available only for the NLSY-79 cohort because the NLSY-97 cohort is too young). Notably, these are cumulative measures of all student debt accrued for enrollment in higher education, prior to taking into account any repayment.^2^ These differ from some commonly used measures in the NLSY such as “current debt” at age 35, which would be the amount remaining to be repaid at age 35. Given prior research on Black borrowers’ relatively high rates of default (Scott-Clayton & Li, 2016), we might expect outstanding student loan balances at age 35 (which take into account repayment or lack thereof) to be more even more stratified than what we show here.

Outcomes measured at the household level are adjusted for the number of household members and economies of scale (i.e., divided by the square root of household size). Research shows that household size adjustments are especially important when making intersectional comparisons, in light of racial and gender differences in factors such as marriage and non-marital partnering, number of resident children, and extended family households (Marsh et al., 2007). All dollar amounts are adjusted to 2017 dollars, corresponding to our most recent data.

We measure all outcomes (with one exception) at age 35 because this is the oldest age at which we can make comparisons across cohorts.^3^ In the most recent data release, most of the NLSY-97 respondents were in their mid-thirties. In addition, most respondents (though not all; Denice, 2017) will have completed their education by age 35. This helps ensure that we have reasonably proxied lifetime educational attainment as well as all student debt respondents accrue for their own education. Research shows that income measured at this age is a reasonable proxy for lifetime earnings (Haider & Solon, 2006). Wealth, however, typically continues accumulating from the age we observe through retirement, when it is spent down; events such as marriage, divorce, and disability also influence wealth trajectories. Prior research shows that wealth trajectories, exposure to trajectory-altering events, and these events’ correlations with wealth trajectories all vary by race and race-gender (Goda & Streeter, 2021). In particular, scholars have highlighted Black women’s high likelihood of trajectory-altering events in mid-to-late life (Addo & Lichter, 2013; Brown, 2012). Our analyses, therefore, capture Black and White women’s and men’s wealth after some of these potential events, but prior to others that may be consequential. In an exception to the age 35 measurement period, we capture student loans accrued for children up through the most recent data release, regardless of respondent age. We do this to account for the wide possible time horizon in terms of when respondents could be investing in children’s education (and because there is no need to cap respondent ages in order to make comparisons to the NLSY-97).

We use median regressions, logistic regressions, or multinomial logistic regressions, depending on the form of the outcome variable and as specified in the figures. We include our main predictor variables in regressions—including race-gender and measured achievement, which is strongly correlated with students’ likelihood of college attendance—but otherwise show descriptive results in order to assess respondents’ educational and economic circumstances as observed.

## Results

### Educational Attainment

**Fig. 1:**
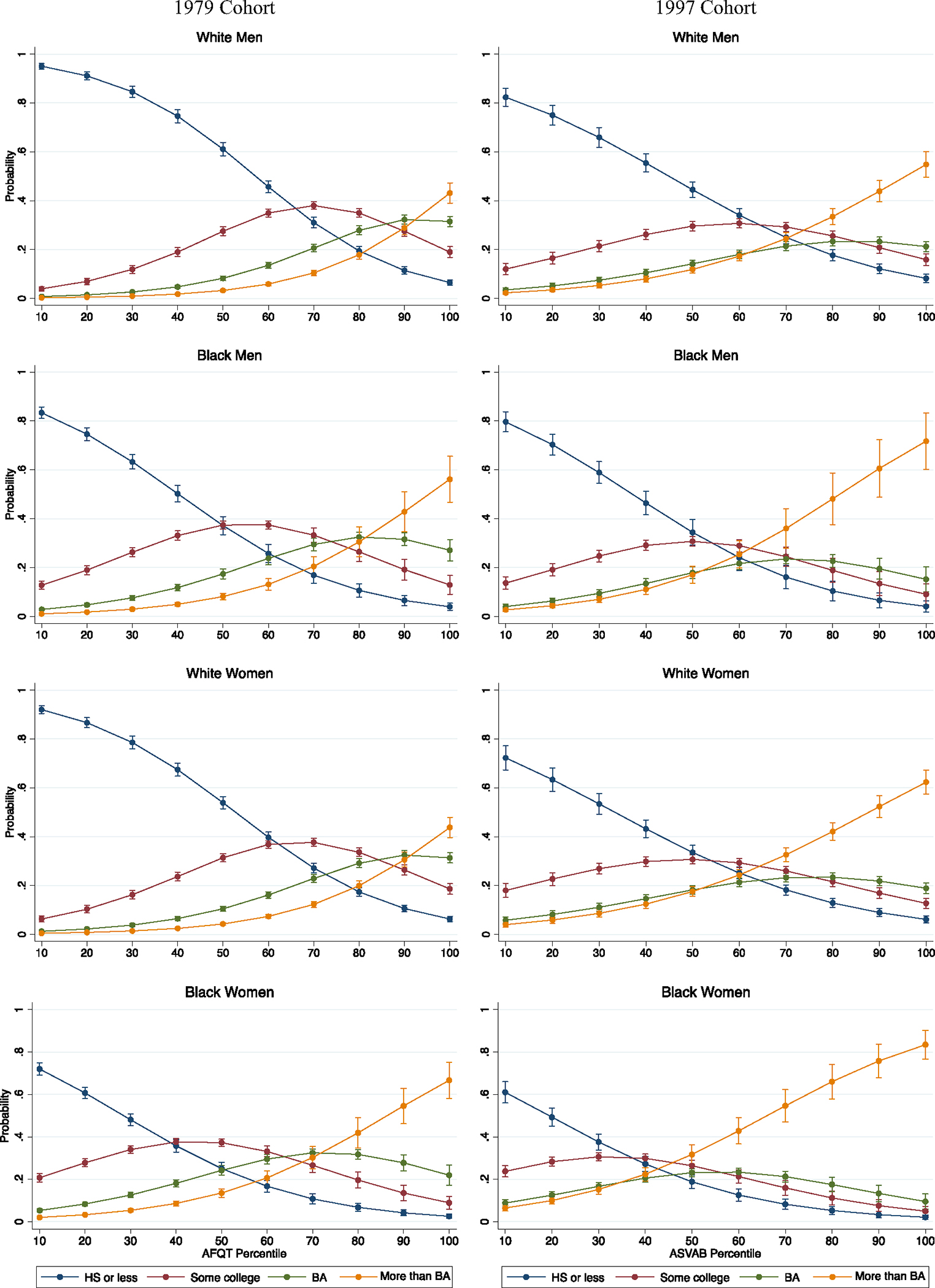
Predicted probability of attaining a given level of education by age 35. Note: Multinomial logistic regressions; 95% confidence intervals shown

We begin by considering race-gender differences in educational attainment across the range of respondents’ test scores and across cohorts. These results are shown in Fig. 1. Given the expansion of higher education that took place in the latter half of the 20th century, we would expect educational attainment to increase across cohorts. This is indeed what we find, although the magnitudes of these increases are larger for some race-gender groups than others. White men, for example, historically have enjoyed relatively unrestricted access to higher education (DiPrete & Buchmann, 2013), and thus it follows that the differences in educational attainment we observe across cohorts (conditional on test scores) are not enormous. Across cohorts, White men at the median of test scores were about equally likely to attain some college or more. White men with the highest test scores became more likely to attain more than a BA across cohorts—from about a 0.45 predicted probability among Baby Boomers, to about a 0.55 predicted probability among Millennials. Changes in Black men’s educational attainment also are relatively modest across cohorts. The patterns observed for the bottom 40 percent of the test score distribution, in particular, are highly consistent for Black men Baby Boomers and Millennials. Like what we saw for White men, Black men with the highest test scores became more likely to attain more than a BA over time, moving from about a 0.55 predicted probability to about 0.75—significantly higher than comparable White men in both cohorts.

Perhaps unsurprisingly given research on the “rise of women” in higher education (DiPrete & Buchmann, 2013), the largest shifts across cohorts are among White and Black women. The highest-achieving White women Baby Boomers attended college fairly often, but even for those at the 80th percentile of test scores, the most likely level of attainment was only “some college.” For White women Millennials, on the other hand, post-graduate education is the most likely outcome at and above the 70th percentile of test scores. Similarly, Black women’s educational attainment, conditional on achievement, is relatively high in both cohorts; even among Baby Boomers, the highest-achieving Black women had about a 0.70 predicted probability of attaining more than a BA. But among Black women Millennials, even for those with only median test scores, the most likely outcome is to proceed beyond the BA. The highest-achieving Black women are virtually guaranteed to attain at least some graduate-level education. Even the lowest-achieving Black women have more than a 0.20 predicted probability of attaining some college, and their predicted probabilities of attaining a BA or more than a BA are both about 0.10. Thus, while all race-gender groups’ educational attainment has shifted upward to some extent across cohorts (conditional on achievement), this was true especially for Black women and, to a slightly lesser extent, White women.

### Household Income

**Fig. 2:**
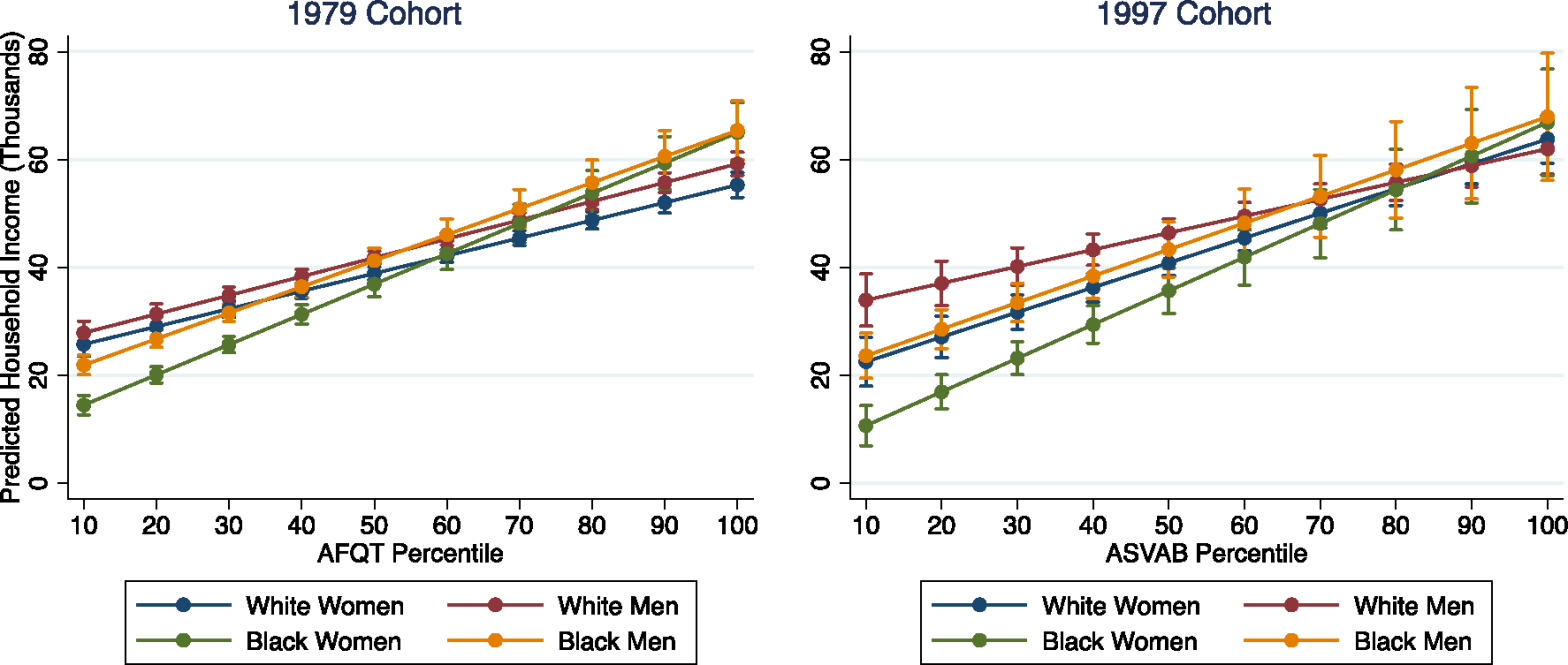
Adjusted household income at age 35. Note: median regressions; 95% confidence intervals shown

Figure 2 shows race-gender differences in median household income across the range of test scores, adjusted for inflation, household size, and economies of scale. Looking across cohorts, we observe patterns of household income that are more similar than they are different. All four racegender groups experience positive returns to achievement in both cohorts, such that higher-achieving respondents have higher household incomes than lower-achieving respondents. Among Baby Boomers, the highest-achieving White women tended to have lower median household incomes than other race-gender groups—significantly lower than White men (*p* < 0.05), Black women (*p* < 0.01), and Black men (*p* < 0.001). Among Millennials, however, these gaps are effectively closed, such that the highest achievers in each race-gender group are not statistically distinguishable in terms of their household income. (Yet, readers should keep in mind that high-achieving members of these racegender groups have different educational attainment that we have not controlled for—so, for example, Black women and White men may have comparable median household incomes conditional on achievement, but Black women at this level of achievement are more likely to have attended graduate school and incurred the costs to do so, as we saw in Fig. 1.)

The most notable shifts across cohorts are concentrated at the bottom of the achievement distribution. In both cohorts, the lowest-achieving Black women have lower household incomes than members of other race-gender groups, but this gap is most prominent in the Millennial cohort. This is partly because the lowest-achieving White men’s household incomes are relatively high, with an adjusted median of about $34,000/year for those at the 10th percentile of achievement (versus about $11,000/year for similarly situated Black women; *p* < 0.001). White men ultimately experience the flattest slope across the range of achievement, but because their intercept is so high, there is no point at which White men are significantly disadvantaged relative to any other race-gender group.

### Household Wealth

**Fig. 3:**
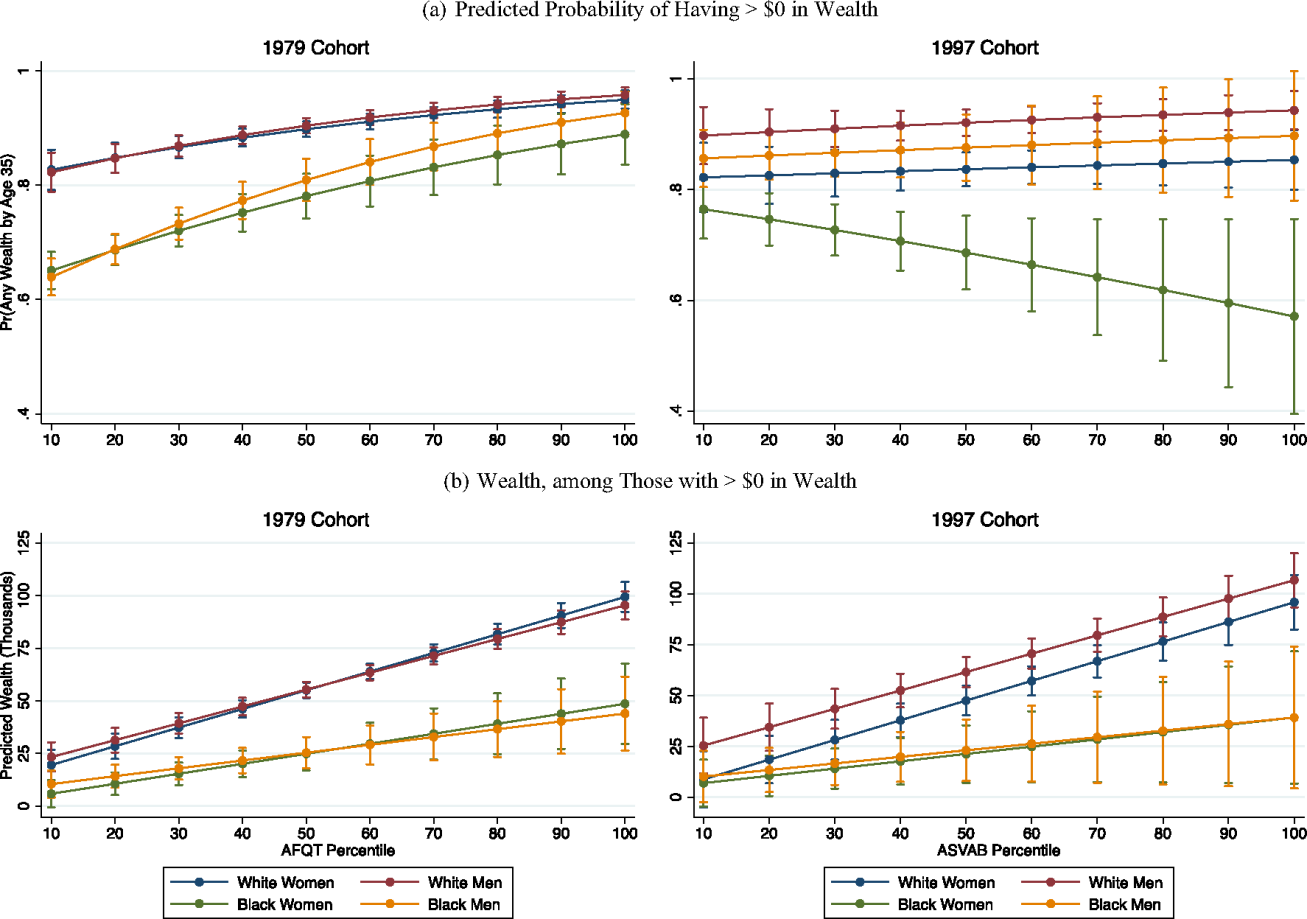
Adjusted household wealth at age 35. *Note*: Logistic regressions in top panel; median regressions in bottom panel; 95% confidence intervals shown

In Fig. 3, we begin to see the contours of how race-gender inequality has deepened across cohorts. The top panel shows respondents’ predicted probability of accumulating greater than $0 in wealth—i.e., any wealth, of any amount greater than zero—at the household level by the time they reach age 35. In the Baby Boomer cohort, as seen in the top-left panel, we observe large racial disparities particularly at the bottom of the achievement distribution, with greater relative gender parity within racial groups. Among those at the 10th percentile of test scores, White men’s and women’s households were about equally likely to hold greater than $0 in wealth by the time they reached age 35, both with a predicted probability of about 0.83. In contrast, Black women’s predicted probability was 0.65, and Black men’s was about 0.64 (both *p* < 0.001 compared to both White men and women). White men’s and women’s chances of having positive wealth continue to increase modestly across the range of test scores, resulting in predicted probabilities of 0.96 for White men and 0.95 for White women with the highest achievement. The highest-achieving Black men are statistically indistinguishable from White men and women, with a predicted probability of 0.93. However, the highest-achieving Black women’s chances of accumulating any wealth at the household level are only about 0.89—significantly lower than White men (*p* < 0.05) and White women (*p* < 0.05) but comparable to Black men.

The bottom panel shows estimates for median household wealth among those with positive wealth. Among those with any household wealth in the Baby Boomer cohort, in the bottom-left panel, we again observe large racial disparities coupled with greater relative gender parity within racial groups. Additionally, these racial gaps widen across the range of achievement. The largest gap in terms of sheer point estimates, for example, is between the highest-achieving White women’s households (adjusted median of about $99,000) and the highest-achieving Black men’s households (adjusted median of about $44,000; *p* < 0.001).

Data from the Millennial cohort tell a different story. As shown in the top-right panel, White men’s, Black men’s, and White women’s households were about equally likely to accumulate wealth across the range of achievement. We also find significant contrasts between White men’s and women’s households, such that White men’s households were more likely to accumulate wealth across virtually the entire range of achievement (except for those with the very lowest test scores; all others at least *p* < 0.05). Yet, we observe a unique pattern among Millennial Black women, such that their chances of accumulating any wealth drop steeply across the range of achievement. Black women at the 10th percentile of test scores have about a 0.77 predicted probability of accumulating any wealth at the household level by age 35—significantly lower than White men’s (*p* < 0.001) and Black men’s (*p* < 0.05) households, despite relatively equal household incomes at this point in the achievement distribution (see Fig. 2). For Black women with the highest test scores, their households’ chances of holding positive wealth are only about 0.57. Put differently, and to reiterate, only about half of the highest-achieving Black women Millennials have any wealth at the household level by age 35. This is by far the lowest point estimate across race-gender groups and across cohorts.

The bottom-right panel, which shows estimates for median wealth among Millennials (conditional on holding any wealth), is patterned similarly to what we saw in the Baby Boomer cohort. The largest disparities are again between racial groups, and these gaps widen across the range of respondents’ test scores. In an exception, we find that White men’s households had greater wealth than White women’s households at moderate levels of achievement, between about the 30th and 70th percentile of test scores (all at least *p* < 0.05). We otherwise observe relative gender parity within the context of large racial gaps in wealth.^4^

### Student Debt

**Fig. 4:**
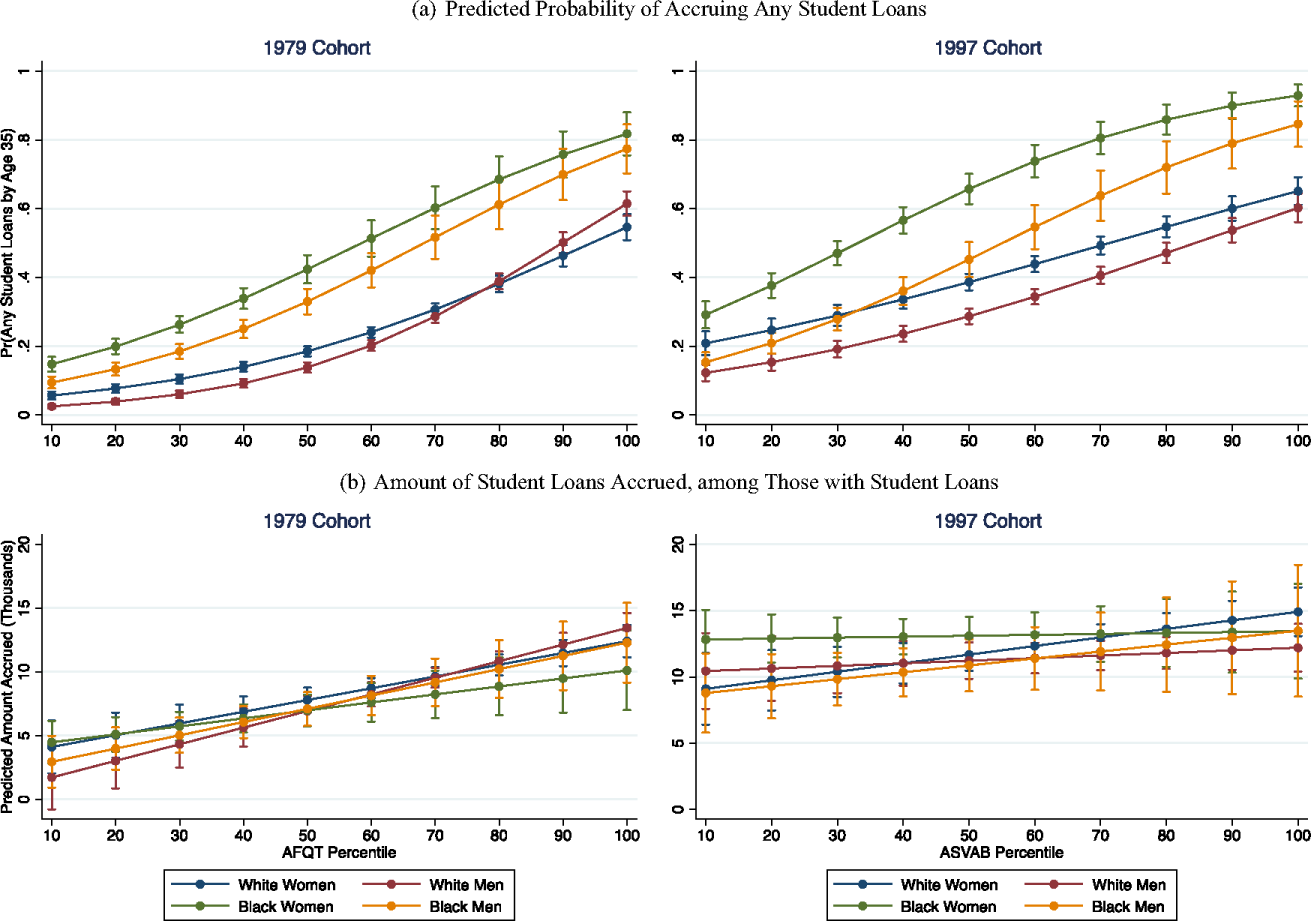
Student loans accrued by age 35. Note: logistic regressions in top panel; median regressions in bottom panel; 95% confidence intervals shown

We now turn to considering the extent to which the incidence and amounts of student debt have changed across cohorts for Black and White women and men. Figure 4 summarizes these results, showing respondents’ predicted probabilities of accruing any student loans by age 35 (top panel) as well as the cumulative amount of debt among those with any student loans (bottom panel). We lift out three patterns that are particularly consequential:

First, as with the results for wealth that we saw in Fig. 3, we observe large racial disparities, such that Black women and men generally are more likely to hold student debt than White women and men. However, unlike what we saw with wealth, we find clear distinctions in terms of gender as well, such that Black women generally are more likely to hold student debt than Black men, and White women generally are more likely to hold student debt than White men. Black women clearly have the highest incidence of debt in both cohorts, but especially in the Millennial cohort. Among NLSY-97 respondents at the median of test scores, for example, Black women’s predicted probability of holding student debt was 0.66, versus 0.45 for Black men, 0.39 for White women, and 0.29 for White men (all comparisons *p* < 0.001 relative to Black women). These disparities, of course, are partly driven by the fact that Black women Millennials with median test scores have attained much more education than their counterparts in other race-gender groups, as we saw in Fig. 1. But even among the highest-achieving respondents, who are all virtually guaranteed to attain at least some higher education, Black women still have the highest incidence of student debt. Nearly all Black women with the highest test scores are expected to accrue student debt by age 35, with a predicted probability of 0.93, versus 0.85 for Black men (*p* < 0.05), 0.65 for White women (*p* < 0.001), and 0.60 for White men (*p* < 0.001).

Second, across cohorts, the incidence of student debt has been lifted upward for most race-gender groups at most points in the test score distribution. This is true especially for White and Black women. For example, among Baby Boomers, White women with median test scores had a 0.20 predicted probability of accruing student debt. In the Millennial cohort, this doubles to approximately 0.40. The upward shift for Black women with median test scores is smaller by comparison—from approximately 0.42 in the Baby Boomer cohort to 0.65 in the Millennial cohort. Yet, considering that Black women have the highest incidence of student debt at most points in the test score distribution in both cohorts, it is striking that their incidence has continued to grow at a high rate across cohorts. White men are something of an exception, especially at the very top of the distribution, where their probability of accruing student loans remains stable across cohorts. Yet, we do see an upward shift among White men with the lowest test scores. Their predicted probability of holding debt is near-zero among Baby Boomers, as compared to 0.12 among Millennials.

**Fig. 5:**
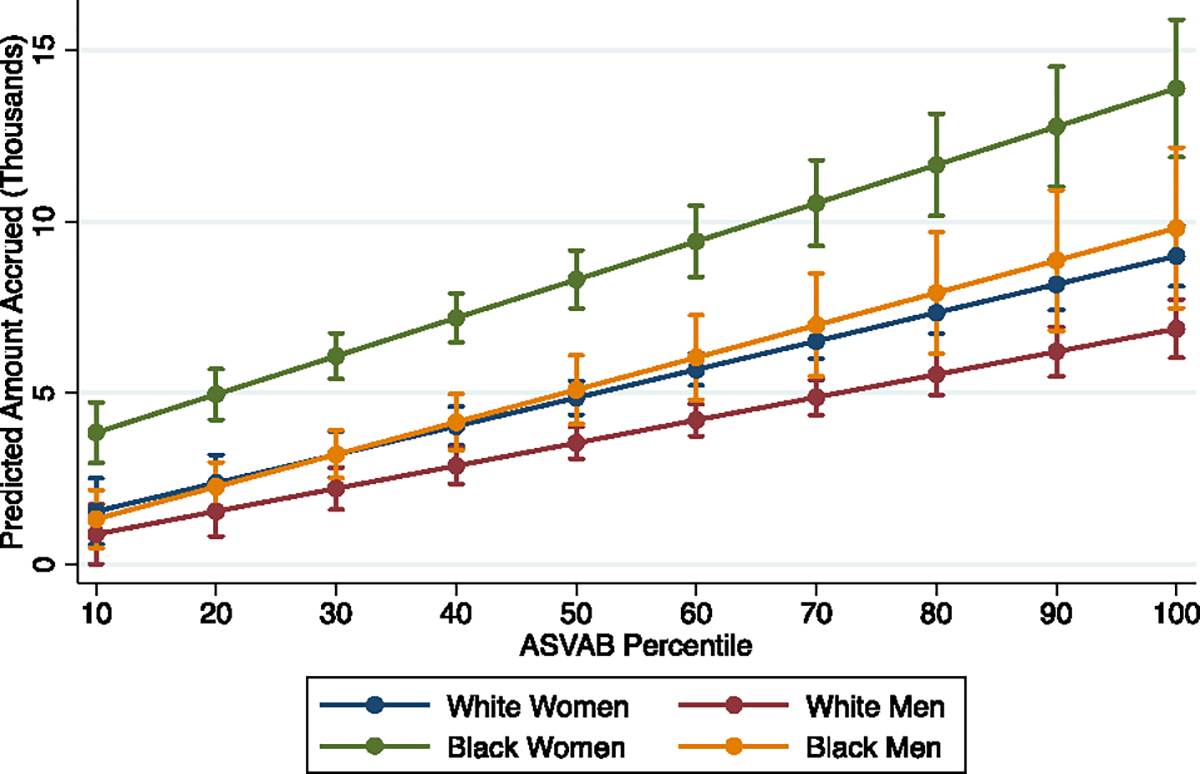
Student loans accrued by age 35—NLSY-97 cohort—inclusive of those with $0 in student loans. Note: median regression; 95% confidence intervals shown

Third, the amounts of debt accrued (conditional on accruing any debt) have stayed relatively consistent across cohorts for two groups: White women and Black men. We observe some minor shifts upward across the distribution of test scores, but the slopes and general patterning are similar for Baby Boomers and Millennials in both race-gender groups. For the other two race-gender groups—Black women and White men—their amounts of student debt in the Millennial cohort (conditional on accruing any debt) are relatively constant across the distribution of test scores, which is distinctly not what is observed among Baby Boomers. With regard to Black women in particular, this pattern is consistent with research we discussed earlier on the predatory inclusion of lower-resourced Black women at for-profit colleges (Cottom, 2017; Seamster & Charron-Chénier, 2017). These students frequently do not complete their degree programs, and even if they do, they experience weak or non-existent economic returns; this is consistent with the patterns of education, income, and debt we observe for Black women Millennials with the lowest test scores. In contrast, rising debt among low-achieving White men is not necessarily a pattern that has been highlighted in the literature. Although the amount of debt may be relatively high among this group, recall that in the top panel, the incidence of student debt for low-achieving White men is very low. In fact, when we consider the median amount of debt among Millennials, inclusive of those with zero values—as shown in Fig. 5—we see that low-achieving White men’s debt does not come close to approaching that of low-achieving Black women.

**Fig. 6:**
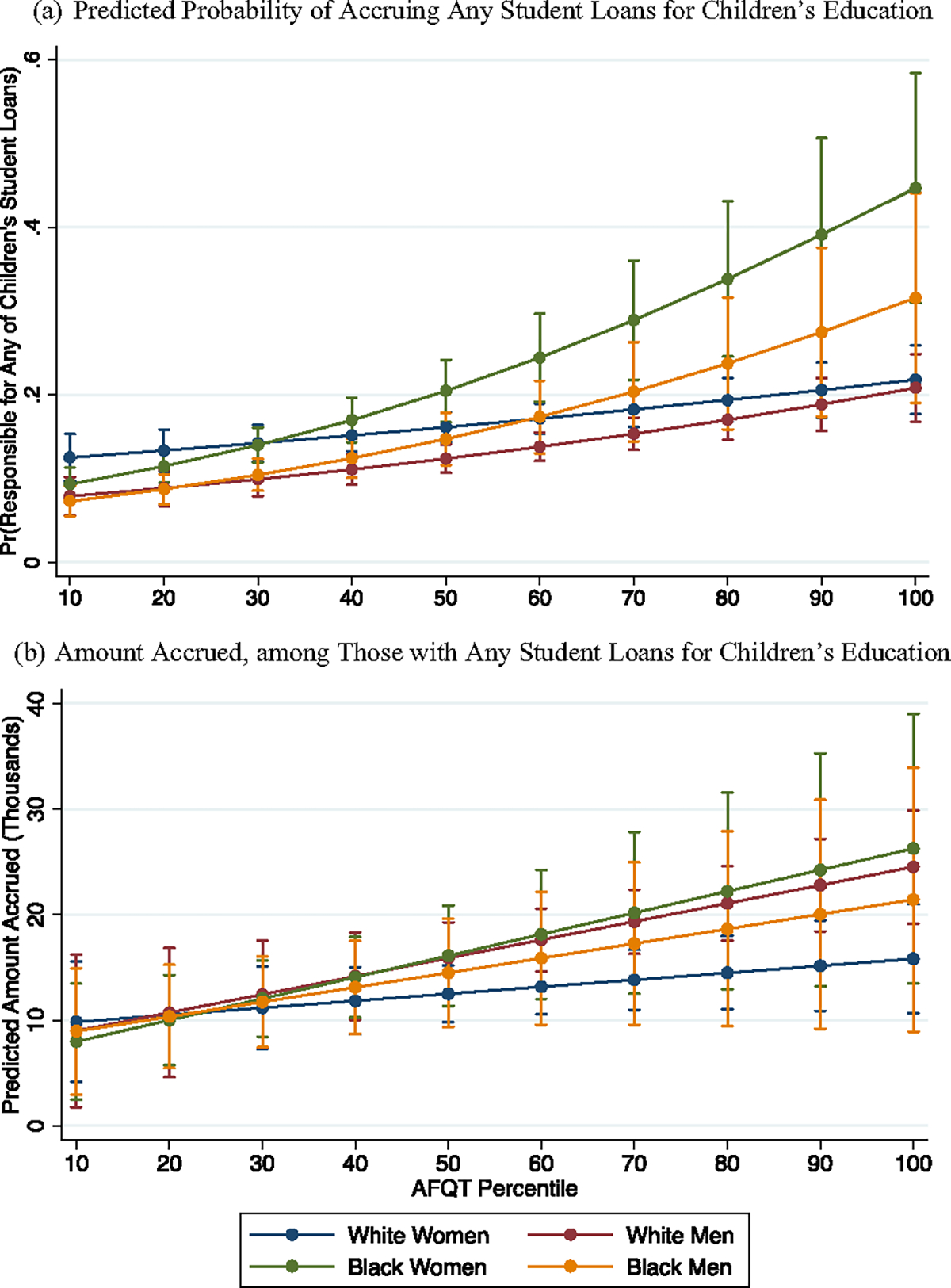
Student loans accrued for children’s education, NLSY-79 cohort. Note: logistic regression in top panel; median regression in bottom panel; 95% confidence intervals shown

Aside from student loans respondents accrue for their own education, another consideration relevant to families’ financial lives is *student loans respondents accrue for their children’s education*. As this point, only the NLSY-79 respondents are old enough to have a critical mass of children who have attended higher education, and thus we restrict our attention to the Baby Boomer cohort for these analyses. Figure 6 shows respondents’ predicted probability of accruing student loans for their children’s education (top panel) as well as the median amount accrued among those with any of their children’s debt (bottom panel). Here, Black women again emerge as uniquely overexposed to educational debt, this time in an intergenerational fashion. As shown in the top panel, the highest-achieving Black women have the highest point estimate of all, with a 0.45 predicted probability of accruing student debt for their children’s education (*n.s.* compared to Black men but *p* < 0.01 compared to White women and men). In the bottom panel, significant differences are much harder to parse, in part owing to a smaller sample size and wide confidence intervals. The highest-achieving Black women have the highest point estimate in this figure, with median debt of about $26,000 (conditional on accruing any student loans for children), although this amount is not significantly higher than that of other racegender groups.

**Fig. 7:**
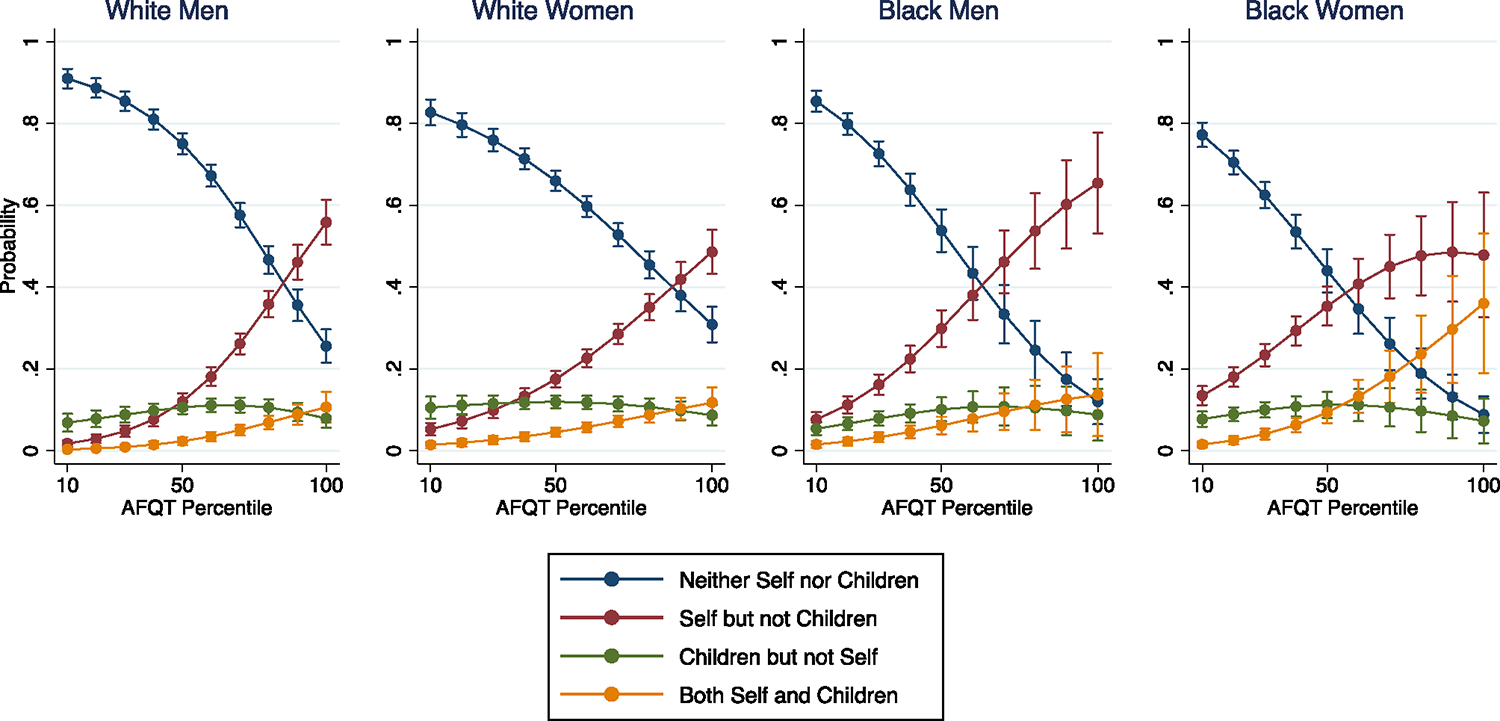
Incidence of student loans for self and/or children, NLSY-79 cohort. Note: multinomial logistic regression; 95% confidence intervals shown. For each race-gender group, we show the predicted probability of respondents accruing student loans for: (1) neither themselves nor their children; (2) themselves, but not their children; (3) their children, but not themslves; and (4) both themselves and their children

Given that our results point to high-achieving Black women’s unique intergenerational exposure to student debt, in Fig. 7, we use a measure that combines data on respondents’ own debt along with their children’s debt. Specifically, we categorize respondents according to whether they: (1) accrued no student debt for themselves or their children; (2) accrued student debt for themselves, but not their children; (3) accrued student debt for their children, but not themselves; or (4) accrued student debt for both themselves and their children. The results reflect multiple dynamics including, but not limited to, respondents’ chances of attending college, their chances of having children, their children’s chances of attending college, and their chances of accruing student debt in their and their children’s interfacing with higher education. But while the mechanisms giving rise to these patterns are complex, they nonetheless clearly capture race-gender disparities in exposure to student debt in intergenerational perspective.

As an illustration of this point, consider those in category (1), who never accrued student debt across the life course. Toward the bottom of the test score distribution, respondents presumably are not accruing debt because they and their children are not attending college. Accordingly, it is not necessarily surprising that these respondents have not accrued any student debt across the life course. Toward the top of the test score distribution, however, respondents (and their children, if they have any) are virtually guaranteed to attend college—thus, race-gender disparities in this location reflect respondents’ multi-generational ability to pay for higher education out of pocket, without having to accrue any debt either as a student or as a parent.^5^ Indeed, we find that the highest-achieving White men (0.26) and White women (0.31) are much more likely to never accrue student debt, relative to their Black men (0.12) and Black women (0.09) counterparts (all comparisons between White and Black respondents *p* < 0.001).

Category (4) similarly requires a dynamic interpretation—these are respondents who accrued debt both as a student and as a parent. Again, toward the bottom of the test score distribution, respondents presumably are not accruing debt because they and their children are not attending college. But toward the top of the test score distribution, respondents and their children presumably *are* attending college, and thus race-gender disparities in this location reflect respondents’ multi-generational exposure to student debt. This is where Black women emerge as overexposed relative to other race-gender groups. Their predicted probability of accruing student loans for both themselves and their children is 0.36—far higher than that of Black men (0.14; *p* < 0.05), White women (0.12; *p* < 0.01), and White men (0.11; *p* < 0.01). Thus, more than any other race-gender group at any other location in the distribution of test scores, the highest-achieving Black women Baby Boomers have been uniquely likely to continually interface with student loans across the life course.

## Conclusion

Using data from Black and White women and men in two recent cohorts, this article has considered how similarly situated students (as measured by their achievement in adolescence) fared in terms of educational attainment and economic outcomes mostly assessed at age 35. These are two cohorts that, despite being born only a few decades apart, experienced vastly different landscapes in terms of higher education opportunity, cost, and access to credit. While the analyses reveal multiple contours in how students and families have interfaced with higher education across the 20th century, some of the most striking findings pertain to high-achieving Black women’s structural overexposure to student debt. Our findings indicate that student debt is a near-modal life course experience for high-achieving Black women, and that intergenerational experiences with student debt also are highly common. We see this as an example of how structural inequalities by both race and gender harm those who, by virtue of ability and opportunity, are best poised for economic mobility through education. This is a trend that has been demonstrated through research on how racial inequalities “harm the best” in K-12 education (Hanushek & Rivkin, 2009; Riegle-Crumb & Grodsky, 2010) but that has not, to our knowledge, been extended into research on higher education and the economic life course. Future research should continue to examine these processes, and especially should follow the Millennial cohort as they approach mid-life and the “student debt generation” potentially accrues yet more debt for their children’s education.

## Figures and Tables

**Table 1:** Distributional data on achivement test scores, by cohort and race-gender group

Race-gender group	n	Achievement test scores		

		25th percentile	50th percentile	75th percentile

NLSY-79: trailing-edge baby boomers (AFQT)				
White men	3541	28.31	53.76	77.78
White women	3502	31.15	53.19	75.36
Black men	1524	7.59	18.02	36.06
Black women	1504	8.88	19.49	36.51
NLSY-97: early millennials (ASVAB)				
White men	1985	32.54	57.73	80.26
White women	1871	36.88	60.09	80.11
Black men	879	7.81	19.44	40.01
Black women	929	10.97	24.85	47.58

## Data Availability

The National Longitudinal Surveys of Youth are publicly available through the Bureau of Labor Statistics: https://www.bls.gov/nls/.

## Footnotes

1 For the student debt analyses, a consequence of our comprehensive sampling strategy is that some respondents accrue “0” in debt because they did not attend college, while others accrue “0” while attending college because their family can pay out of pocket. We also analyze amounts of student debt among those with any debt, which necessarily restricts this sample to college attendees. Both of these samples and the implications of these samples are discussed in the results.

2 For example, if a student accrued $10,000 for each of four years of undergraduate education, their amount of student debt accrued at age 35 would be $40,000, even if they had already paid back their loans in full.

3 If respondents’ economic outcomes were missing at age 35, we flled them in with amounts reported at ages 36–40 as available; for the NLSY-97 cohort, the very oldest respondents were 38 in our data.

4 When we use respondents’ relative place in the wealth distribution as an outcome rather than specific dollar amounts, the look and patterning of results is consistent with what we show in the main text. The analyses using wealth percentiles tend to place greater emphasis on gaps between Black men and women in the Millennial cohort, compared to what we show here.

5 Based on the NLSY coding scheme, if a respondent’s child accrued student debt in their name, respondents could still be sorted into category (1) as long as they did not accrue any parent loans. We suspect this is relatively uncommon among those with the highest test scores in category (1), but it is a possibility nonetheless.
